# Extra-striatal D_2/3_ receptor availability in youth at risk for addiction

**DOI:** 10.1038/s41386-020-0662-7

**Published:** 2020-04-07

**Authors:** Natalia Jaworska, Sylvia M. L. Cox, Maria Tippler, Natalie Castellanos-Ryan, Chawki Benkelfat, Sophie Parent, Alain Dagher, Frank Vitaro, Michel Boivin, Robert O. Pihl, Sylvana M. Côté, Richard E. Tremblay, Jean R. Séguin, Marco Leyton

**Affiliations:** 1 Institute of Mental Health Research, affiliated with the University of Ottawa, Ottawa, ON Canada; 2 Department of Cellular & Molecular Medicine, University of Ottawa, Ottawa, ON Canada; 3 Department of Psychiatry, McGill University, Montreal, QC Canada; 4 Montreal Neurological Institute, McGill University, Montreal, QC Canada; 5 School of Psychoeducation, Université de Montréal, Montreal, QC Canada; 6 CHU Ste-Justine Research Center, Montreal, QC Canada; 7 Department of Psychology, Université Laval, Montreal, QC Canada; 8 Department of Psychology, McGill University, Montreal, QC Canada; 9 Department of Social & Preventative Medicine, Université de Montréal, Montreal, QC Canada; 10 Department of Pediatrics & Psychology, Université de Montréal, Montreal, QC Canada; 11 Department of Psychiatry, Université de Montréal, Montreal, QC Canada; 12 Center for Studies in Behavioral Neurobiology, Concordia University, Montreal, QC Canada

**Keywords:** Addiction, Risk factors

## Abstract

The neurobiological traits that confer risk for addictions remain poorly understood. However, dopaminergic function throughout the prefrontal cortex, limbic system, and upper brainstem has been implicated in behavioral features that influence addiction vulnerability, including poor impulse control, and altered sensitivity to rewards and punishments (i.e., externalizing features). To test these associations in humans, we measured type-2/3 dopamine receptor (DA_2/3_R) availability in youth at high vs. low risk for substance use disorders (SUDs). In this study, *N* = 58 youth (18.5 ± 0.6 years) were recruited from cohorts that have been followed since birth. Participants with either high (high EXT; *N* = 27; 16 F/11 M) or low pre-existing externalizing traits (low EXT; *N* = 31; 20 F/11 M) underwent a 90-min positron emission tomography [^18^F]fallypride scan, and completed the Barratt Impulsiveness Scale (BIS-11), Substance Use Risk Profile scale (SURPS), and Sensitivity to Punishment (SP) and Sensitivity to Reward (SR) questionnaire. We found that high vs. low EXT trait participants reported elevated substance use, BIS-11, SR, and SURPS impulsivity scores, had a greater prevalence of psychiatric disorders, and exhibited higher [^18^F]fallypride binding potential (BP_ND_) values in prefrontal, limbic and paralimbic regions, even when controlling for substance use. Group differences were not evident in midbrain dopamine cell body regions, but, across all participants, low midbrain BP_ND_ values were associated with low SP scores. Together, the results suggest that altered DA_2/3_R availability in terminal extra-striatal and dopamine cell body regions might constitute biological vulnerability traits, generating an EXT trajectory for addictions with and without co-occurring alterations in punishment sensitivity (i.e., an internalizing feature).

## Introduction

In many societies, adolescent substance use is the norm. Despite this, only some will develop a substance use disorder (SUD). The best characterized vulnerability traits are diverse externalizing (EXT) features [1, 2], including impulsivity and altered responses to rewards and punishments [3]. These features manifest early and continue throughout adolescence, increasing the probability of developing SUDs and comorbid psychiatric problems [4, 5].

The neurobiology mediating these vulnerability features is not well understood, but both increases and decreases in mesocorticolimbic dopamine (DA) function have been implicated. Most of this work has focused on the striatum [4, 6, 7], but there is growing interest in the contributions of extra-striatal regions. In laboratory animals, prefrontal DA transmission influences temporal discounting [8–10] and most elements of executive function [11]. Dopamine transmission in the amygdala and anterior cingulate, in comparison, increases the willingness to sustain effort to obtain rewards [8, 9], hippocampal DA transmission fosters the formation and activation of emotionally charged memories [12], and DA release in the ventral prelimbic cortex tracks changes in reward rate probability [13].

In humans, research on SUD risk traits in relation to extra-striatal DA has focused on type-2/3 DA receptors (DA_2/3_Rs). A cohesive picture has yet to emerge, but a few relatively small neuroimaging studies raise the possibility that individual differences in impulsivity covary positively with DA_2/3_R availability in the temporal cortex and thalamus [14], while amygdalar DA_2/3_R availability influences emotion regulation [15], and elevated DA_2/3_R levels in the prefrontal cortex (PFC) has been associated with larger amygdala responses to unpleasant stimuli [16]. Negative correlations, in comparison, have been reported between midbrain cell body region DA_2/3_R availability and impulsivity-related personality traits [7], drug cue-induced drug craving [17], and striatal DA release [7, 17]. These latter effects have been proposed to reflect inhibitory feedback from somatodendritic autoreceptors on DA cells that project to the striatum [7, 17]. Indeed, in humans, midbrain DA_2/3_ autoreceptors appear to be largely restricted to mesostriatal neurons [17, 18].

To better test the hypothesized relation between extra-striatal DA_2/3_R availability and pre-existing SUD risk traits, we conducted [^18^F]fallypride positron emission tomography (PET) scans in a relatively large sample of 18–20-year-old youth recruited from research participants who have been characterized and followed since birth, and who exhibited high vs. low EXT traits during early adolescence (between the ages of 10 and 16 years). We predicted that the high vs. low SUD risk participants, based on EXT traits, would have lower [^18^F]fallypride nondisplaceable binding potential (BP_ND_) values in the midbrain, and elevated BP_ND_ values in the PFC and limbic and paralimbic terminal regions, particularly in the amygdala. Individual differences in DA_2/3_R availability would be associated with EXT features, including impulsivity, other substance use risk traits, and sensitivity to reward and punishment.

## Methods and materials

### Participants

We recruited transitional age youth (18–20 year) from: (i) the “Quebec Longitudinal Study of Child Development” (QLSCD; 31 participants from the 572 born in 1996 [19], and 22 from 2120 born in 1997–1998 [20]) and (ii) the “Quebec Newborn Twin Study” (QNTS; 5 participants from 662 twin pairs born in 1995–1997 [21]; only one sibling per pair tested). All participants lived in the area of Montreal and Quebec City, and had been followed since birth.

Eligibility was based on questions from developmentally appropriate behavior questionnaires [22–24], which were used to develop the “social behavior questionnaire” [25]. A subset of the scores, from the first wave of QLSCD study members (*N* = 242), was summed to form an aggregate EXT trait score; i.e., mean scores for the subscales hyperactivity, impulsivity, oppositional behavior, nonaggressive behavioral problems and aggression (proactive, indirect, reactive) [25]. Mean EXT scores during early adolescence correlated with those obtained earlier in life (1–5 and 6–10 years) and predicted substance use at age 16 years [25]. EXT cut-off scores representing individuals that fell in the top and bottom 30%, as established in the first wave of study members, were then applied to the entire sample of participants (i.e., total QLSCD and QNTS samples) with a minimum of two assessments between ages 10 and 16 years. These individuals were considered at high vs low risk for substance use problems, respectively [25], and were invited to participate in the neuroimaging study. The final [^18^F]fallypride imaging sample consisted of 31 low-risk (20 F/11 M) and 27 high-risk trait youth (*N* = 27, 16 F/11 M; Table 1).

**Table 1:** Demographic data and questionnaire scores in high and low externalizing (EXT) trait score youth (means [M] ± standard deviations [SD]).

Feature	High EXT group	Low EXT group	Statistical test	Partial Eta squared (partial η^2^)
*N* (*N* = 58)	27	31	–	–
F/M	16 F/11 M	20 F/11 M	*X*^2^ test: *p* = 0.68	–
Age (yr)	18.59 ± 0.64	18.42 ± 0.56	Univariate ANOVA: *p* = 0.36	0.021
Ethnicity	*N* = 26, White; *N* = 1 Black	*N* = 30, White *N* = 1 Mixed (Hispanic/White)	–	
Education (yr)	11.78 ± 0.80	12.10 ± 1.14	Univariate ANOVA: *p* = 0.23	0.026
Externalizing trait scores*	2.40 ± 0.58	0.48 ± 0.29	Univariate ANOVA: *p* < 0.001	0.82
AUDIT scores	5.96 ± 4.24	4.00 ± 2.63	Univariate ANOVA: *p* = 0.036	0.076
Age of alcohol use onset	14.09 ± 2.31	15.15 ± 1.95	Univariate ANOVA: *p* = 0.065	0.060
Lifetime occasions of binge drinking	56.11 ± 89.33	35.17 ± 66.84	Univariate ANOVA: *p* = 0.021	0.09
Lifetime occasions of alcohol use (intoxication & non-intoxication)	120.79 ± 149.28	77.00 ± 84.23	Univariate ANOVA: *p* = 0.17	0.033
Lifetime cannabis use	22Y/5 N	19Y/12 N	*X*^2^ test: *p* = 0.092	–
Age of onset of cannabis use	15.5 ± 1.60	16.26 ± 1.19	Univariate ANOVA: *p* = 0.095	
Lifetime occasions (#) of cannabis use	309.19 ± 542.38	32.84 ± 142.00	Univariate ANOVA: *p* = 0.008	0.12
Lifetime occasions (#) of drug use, excluding cannabis**	27.11 ± 88.04	1.06 ± 4.44	Univariate ANOVA: *p* = 0.105	0.046
Lifetime drug use, excluding cannabis	11Y/16 N	4Y/27 N	*X*^2^ test: *p* = 0.004	–
Current tobacco smoking status	3Y/24 N	2Y/29 N	*X*^2^ test: *p* = 0.53	–
Lifetime tobacco smoking	*N* = 13 never smoked; *N* = 8 < 10 in lifetime; *N* = 6 > 11 in lifetime	*N* = 25 never smoked, *N* = 4 < 10 in lifetime; *N* = 2 > 11 in lifetime	*X*^2^ test: *p* = 0.032	–
Likely current and/or past psychiatric disorder history	14 N/13Y	30 N/1Y	*X*^2^ test: *p* < 0.001	–
Likely current psychiatric disorder	18 N/9Y (*N* = 5: SUD; *N* = 1: ADHD; *N* = 1 persistent depressive disorder; *N* = 1: ADHD, anxiety, SUD; *N* = 1 dyslexia [mild])	31 N/0Y	*X*^2^ test: *p* < 0.001	–
SURPS Impulsivity	*N* = 25, 11.48 ± 2.90	*N* = 31, 8.97 ± 2.57	Univariate ANOVA: *p* = 0.001	0.18
SURPS Hopelessness	12.96 ± 3.31	11.35 ± 2.74	*p* = 0.071	0.059
SURPS Anxiety sensitivity	10.20 ± 2.29	9.48 ± 3.10	*p* = 0.34	0.017
SURPS Sensation seeking	16.64 ± 3.60	16.32 ± 4.24	*p* = 0.77	0.002
BIS Attention	*N* = 25, 16.28 ± 2.98	*N* = 31, 14.13 ± 3.36	Univariate ANOVA: *p* = 0.015	0.10
BIS Motor	21.72 ± 4.08	18.29 ± 3.08	*p* = 0.001	0.19
BIS Nonplanning	25.36 ± 3.88	22.03 ± 4.17	*p* = 0.003	0.15
BIS Total	63.36 ± 8.13	54.45 ± 7.75	*p* < 0.001	0.25
SPSRQ Reward	N = 26, 10.73 ± 4.52	*N* = 30, 8.53 ± 3.01	Univariate ANOVA: *p* = 0.035	0.08
SPSRQ Punishment	11.96 ± 5.06	9.57 ± 4.82	*p* = 0.076	0.057
SPSRQ Reward/Punishment ratio	1.14 ± .90	1.17 ± .84	*p* = 0.91	0.001

*AUDIT* alcohol use disorders identification test, *BIS-11* Barratt impulsiveness scale, *SPSRQ* sensitivity to punishment and sensitivity to reward questionnaire, *SUD* substance use disorder, *SURPS* substance use risk profile scale.

*Cut-off EXT scores were <0.95 for the low EXT group and >1.77 for the high EXT group.

**Number of occasions of lifetime drug use for non-medical purposes (excluding cannabis and cigarettes).

### Screening and clinical information

Cohort members who expressed interest in the present study were telephone screened. Eligible participants were then invited for in-person assessments using the Structured Clinical Interview for DSM-5 [26]. All provided drug and alcohol use histories using the Timeline Follow-Back Method [27] and Alcohol Use Disorders Screening Test (AUDIT) [28], supplementing data that had been collected prospectively during annual interviews between ages 10 and 16 years. Participants also completed the Substance Use Risk Profile Scale (SURPS) [29], Barratt Impulsiveness Scale (BIS-11) [30], and Sensitivity to Punishment (SP) and Sensitivity to Reward (SR) Questionnaire (SPSRQ) [31]. The scales have demonstrated validity in adolescents and young adults, and have acceptable test-retest reliability [32–34]. Ethics approval was granted by the McGill University and Sainte-Justine University Hospital Research Ethics Boards, and all participants provided written informed consent.

Participants were excluded if they were currently taking psychotropic medication, had magnetic resonance imaging (MRI) or PET contraindications or significant medical conditions (Table 1). Females were excluded if they were pregnant (urine test: Biostrip HCG, Innovatek Medical Inc., Delta, BC, Canada).

Urine drug screens (Express Diagnostics, MN, USA) were obtained prior to PET scans, and participants were excluded if they tested positive for drugs (amphetamine, benzodiazepines, buprenorphine, cocaine, 3,4-methylenedioxy-methamphetamine, methamphetamine, methadone, opioids) other than tetrahydrocannabinol (THC). Five participants tested positive for THC but were not acutely intoxicated, and were included in the study (all in the high EXT group; Table 1). Participants were asked to refrain from caffeine for >4 h, nicotine for >12 h (one participant abstained for only 6.5 h; excluding this individual did not alter our results, presented below), and alcohol for >24 h prior to their PET scan. A breathalyser test ensured alcohol abstinence (BACtrack S80, KHN Solutions LLC, CA, USA). To control for gonadal hormone fluctuations, females who were not using a hormonal contraceptive were scanned during their follicular phase (days 1–10, self-report); all participants were scanned within a narrow time window (13h00–16h30).

### Neuroimaging

#### Magnetic resonance imaging (MRI) and positron emission tomography (PET) acquisition

MRI scans were acquired using a 3 T Siemens Trio TIM scanner (McConnell Brain Imaging Centre, Montreal Neurological Institute) with a Magnetization Prepared Rapid Acquisition sequence (slice: 1 mm; TR: 2300 ms, TE: 3.42 ms, flip angle: 9°, FOV: 256 mm, Matrix: 256 × 256).

PET scans were acquired with a high-resolution research tomograph (HRRT, CTI/Siemens). Following cannula insertion into the left antecubital vein for tracer administration, a 6-min ^137^Cs transmission scan was obtained for attenuation correction. The [^18^F]fallypride tracer (prepared as previously described [17]) was administered as a 1-min intravenous bolus, with emission scans acquired concurrently in list mode over 90-min (participants were instructed to remain awake). The average [^18^F]fallypride dose was 3.33 ± .20 mCi (3.1–3.7 mCi; **~**125MBq), and specific activity was 6929.2 ± 6688 GBq/µmol (no group differences, *p* > 0.10). Previous work indicates that this corresponds to an effective dose equivalent of [^18^F]fallypride of ~0.021 mSv/MBq [35], thus, participants were exposed to ~2.6 mSv, on average. There were no group differences in PET tracer dose (low EXT: 3.28 ± 0.23 mCi [121.4MBq]; high EXT: 3.38 ± 0.14 mCi [125.06MBq]). Prior to the [^18^F]fallypride scan, all participants had a 60-min PET scan with a [^11^C]-labeled tracer [36].

PET images were reconstructed using the Ordinary Poisson Ordered Subset Expectation Maximization reconstruction algorithm (10 iterations, 16 subsets). This included correction for nonuniformities, attenuation, scattered and random coincidences, and motion. To reduce partial volume effects, resolution modeling using the point spread function was implemented in image reconstruction. Motion correction was based on a data-driven motion estimation and correction method that estimates rigid-body motion between dynamic frames [37]. Reconstructed image frames consisted of 256 × 256 × 207 voxels (voxel length = 1.21875 mm).

#### MRI and PET analyses

MRIs were pre-processed with the CIVET 2.0.0 pipeline (wiki.bic.mni.mcgill.ca/ServicesSoftware/CIVET), which included correction for image intensity and nonuniformity, and nonlinear and linear transformations to standardized stereotaxic space using the ICBM template [38]. Normalized images were then classified into white matter, gray matter and cerebral spinal fluid, and segmented using a probabilistic atlas based approach (Automatic Nonlinear Image Matching and Anatomical Labeling [ANIMAL]) [39].

Regions of interest (ROIs) were defined on each individual’s CIVET-processed MRI scans using standardized masks (defined on the MNI ICBM-152 template, and registered to each participant’s MRI via linear and nonlinear transformations). Frontal ROIs were defined using ANIMAL segmentation, and included the gray matter of bilateral superior and middle frontal gyri (i.e., dorsolateral PFC aspects), the medial orbitofrontal cortex (OFC) and medial frontal gyri (i.e., medial PFC aspects); these regions were similar to those used in another study by our group assessing extra-striatal DA_2/3_R availability [17]. Limbic and paralimbic ROIs (amygdala, hippocampus, insula) were derived from the Talairach atlas [40] using the Talairach Deamon and PickAtlas software. Finally, a whole midbrain ROI (substantia nigra, ventral tegmental area) was derived from a mask provided by Dr. Adcock’s Laboratory at Duke University (MNI ICBM-152 space) [41]. Striatal DA_2/3_R expression was not measured as scans longer than 90 min are required for this purpose [42].

ROI masks (Supplementary Fig. 1), were applied to each summed PET image using nonlinear coregistration. Time-activity curves were extracted from each ROI in native PET space using tools developed by the Turku PET Centre (http://www.turkupetcentre.net/). BP_ND_ values (i.e., equilibrium ratio of specifically bound to nondisplaceable radioligand in tissue) were derived from ROIs, averaged across the hemispheres, using the simplified reference tissue model [43], with cerebellar gray matter as the reference region.

### Statistical analyses

#### Demographic and clinical data

Groups (high/low EXT) were compared on pertinent variables with univariate analyses of variance (ANOVAs) or Chi-Square tests (Table 1). Multivariate ANOVAs were carried out to assess group differences on the SURPS, BIS-11, and SPSRQ.

#### [^18^F]Fallypride BP_ND_ values

A repeated-measures analysis of covariance (rmANCOVA) was conducted for DA terminal regions with group (high/low EXT) as the between-subject factor and region as within subject-factors (superior frontal gyri, middle frontal gyri, medial frontal gyri, OFC, amygdala, hippocampus, insula). A univariate ANCOVA with group (high/low EXT) as the between-subject factor was carried out for the midbrain. The following covariates were included: number of lifetime cannabis occasions, number of lifetime noncannabis drug occasions, AUDIT scores, and current smoking status (yes/no). These covariates were selected to control for drug use, which may affect [^18^F]fallypride BP_ND_ values, and other DA activity indices [44, 45]. Tests of normality of the ROI BP_ND_ value residuals were assessed using the Kolmogorov–Smirnov test. If normality was violated, BP_ND_ values were examined for outliers, which were removed (>±3 SD group mean). Greenhouse–Geisser corrections were applied when sphericity was violated (*p* < 0.05).

#### Correlations between [^18^F]Fallypride BP_ND_ and clinical measures

Spearman correlations were carried out between variables of interest (SURPS [four factors], BIS [three factors], SPSRQ [two factors]) and ROI BP_ND_ values. Significance was set to *p* < 0.0007 for the correlations (*p* = 0.05/72 [9 questionnaire factors × 8 ROIs]). Partial correlations were secondarily carried out for significant correlations controlling for AUDIT scores, lifetime drug use occasions (all drugs, apart from cannabis, tobacco, alcohol), lifetime cannabis use occasions, and current tobacco smoking status. Correlations were conducted for the entire sample and per group (outliers removed; Table 2).

**Table 2:** Adjusted fallypride BP_ND_ values in regions of interest in high and low externalizing (EXT) trait score youth (means ± standard errors of the mean).

Regions of interest	High EXT group (*N* = 27)	Low EXT group (*N* = 31)	Statistical test*	Partial Eta squared (partial *η*^2^)
Superior frontal gyrus	0.69 ± 0.049	0.53 ± .047	*p* = 0.032	0.085
Middle frontal gyrus	0.85 ± 0.050	0.68 ± .048	*p* = 0.020	0.10
Medial frontal gyrus	0.79 ± 0.045	0.64 ± .043	*p* = 0.029	0.088
Orbitofrontal gyrus	1.06 ± 0.050	0.97 ± 0.049 (*N* = 30; 1 outlier removed)	*p* = 0.24	0.027
Hippocampus	1.51 ± 0.054 (*N* = 26; 1 outlier removed)	1.43 ± 0.053 (*N* = 29; 2 outliers removed)	*p* = 0.28	0.025
Amygdala	3.29 ± 0.11 (*N* = 26; 1 outlier removed)	2.95 ± 0.11 (*N* = 30; 1 outlier removed)	*p* = 0.038	0.088
Insula	1.70 ± 0.069 (*N* = 26; 1 outlier removed)	1.49 ± 0.068 (*N* = 29; 2 outliers removed)	*p* = 0.048	0.081
Midbrain	1.97 ± 0.073 (*N* = 26; 1 outlier removed)	1.83 ± 0.070 (*N* = 28; 2 outliers removed/1 file unavailable)	*p* = 0.18 (univariate ANCOVA)	0.038

Presented means are adjusted for AUDIT scores, current smoker status (yes/no), occasions of cannabis use (lifetime) and occasions of drug use (lifetime; excluding cannabis, alcohol, and cigarettes) included in the repeated-measures ANCOVA.

*Reflect pairwise comparisons (univariate ANCOVA) of interactions with group as a factor (broken down by group) in the repeated measures ANCOVAs (i.e., group × region), regardless of whether the interaction was significant or not.

## Results

### Demographic and clinical data

The high vs. low-risk groups were well-matched on family income (above/below 39,999CDN/year, data not shown), age and sex; by design, they differed on EXT scores [F(1,56) = 262.30, *p* < 0.001; partial *η*^2^ = 0.82]. They also differed on EXT-related features. Compared with the low EXT group, high EXT participants had elevated BIS-11 [Wilk’s Lambda = 5.82 (df = 3,52), *p* = 0.002; partial *η*^2^ = 0.25], SPSRQ [Wilk’s Lambda = 3.41 (df = 2,53), *p* = 0.04; partial *η*^2^ = 0.11] and SURPS scores [Wilk’s Lambda = 3.22 (df = 4,51), *p* = 0.02; partial *η*^2^ = 0.20] (Table 1).

High EXT participants also exhibited evidence of more problematic drinking, as indexed by higher AUDIT scores [F(1,56) = 4.64, *p* = 0.036; partial *η*^2^ = 0.076] and more occasions of binge drinking [F(1,57) = 5.65, *p* = 0.021, *η*^2^ = 0.09], a greater number of lifetime cannabis use occasions [F(1,56) = 7.48, *p* = 0.008; partial *η*^2^ = 0.12], more use of drugs other than alcohol, tobacco and cannabis [Chi = 5.83, (df = 1,58), *p* = 0.004], and an elevated history of psychiatric disorders including mild SUDs, mood and anxiety disorders [Chi = 15.90, (df = 1,58), *p* < 0.001] (Table 1).

### [^18^F]Fallypride BP_ND_ values

**Fig. 1:**
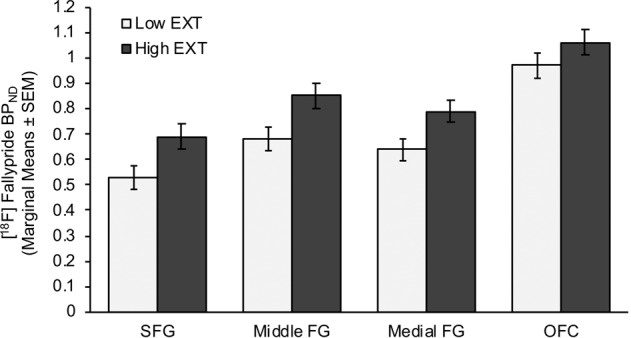
Prefrontal Cortex (PFC): [^18^F]fallypride BP_ND_ values in the superior frontal gyrus (SFG), middle frontal gyrus (Middle FG), medial frontal gyrus (Medial FG) and orbitofrontal cortex (OFC) in the high (High EXT) vs. low (Low EXT) externalizing trait groups. Means are adjusted for alcohol use disorders identification test (AUDIT) scores, current smoker status (yes/no), occasions of cannabis use (lifetime) and occasions of drug use (lifetime; excluding cannabis, alcohol, cigarettes) included in the repeated-measures ANCOVA.

**Fig. 2:**
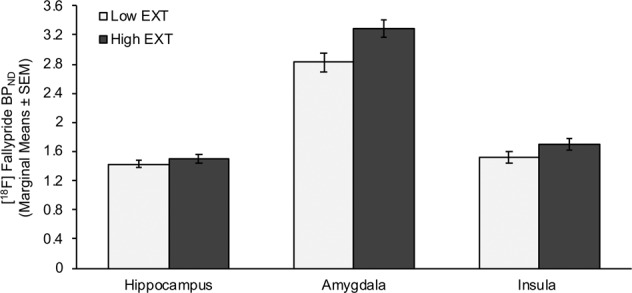
Limbic regions: [^18^F]fallypride BP_ND_ in the amygdala, hippocampus and insula in high (High EXT) vs. low (Low EXT) externalizing trait groups. Means are adjusted for alcohol use disorders identification test (AUDIT) scores, current smoker status (yes/no), occasions of cannabis use (lifetime) and occasions of drug use (lifetime; excluding cannabis, alcohol, cigarettes) included in the repeated-measures ANCOVA.

The rmANCOVA for BP_ND_ values in DA terminal regions yielded a main effect of group [F(1,51) = 4.60, *p* = 0.025; partial *η*^2^ = .10] and region [F(2.31,106.17) = 328.44, *p* < 0.001; partial *η*^2^ = 0.88]. The effect of region reflected highest values in the amygdala followed by the insula, hippocampus, OFC, middle frontal gyrus, medial frontal gyrus, and superior frontal gyrus (Figs. 1, 2; Table 2). The effect of group reflected higher values in high EXT participants. A group × region interaction effect was evident at the trend level [F(2.31,106.17) = 2.55, *p* = 0.074; partial *η*^2^ = 0.053]. Follow-up comparisons confirmed higher adjusted BP_ND_ in the high vs. low EXT group in all regions (*p*s < 0.05) except for the OFC and hippocampus, where the effects were less compelling (*p*s > 0.30). Inclusion of a positive THC screen (*N* = 5) as an additional covariate did not change the pattern of terminal region BP_ND_ results (main effect of group: *p* = 0.009; partial *η*^2^ = 0.15); the same was true of including the presence of past or current SUD as an additional covariate (*N* = 5; main effect of group: *p* = 0.011; partial *η*^2^ = 0.13). Including both a positive THC screen and past or current SUD as additional covariates in the analyses strengthened the main group effect [F(1,41) = 9.02, *p* = 0.005; partial *η*^2^ = 0.18, generally considered a medium effect size]; the group × region interaction continued to be significant at the trend level (*p* = 0.077), Greenhouse–Geisser corrected.

There was no effect of group for midbrain BP_ND_ values [F(1,48) = 1.90, *p* = 0.18; partial *η*^2^ = 0.038; Table 2]. The addition of a positive THC screen (*N* = 5; *p* = 0.18), past or current SUD (*N* = 5, *p* = 0.13) or both features (*p* = 0.13) as additional covariates did not reveal a main effect of group on midbrain BP_ND_ values. Raw BP_ND_ values (i.e., means unadjusted by the inclusion of covariates) per ROI are provided in Supplementary Table 1.

### Correlations between [^18^F]Fallypride BP_ND_ and clinical measures

**Fig. 3:**
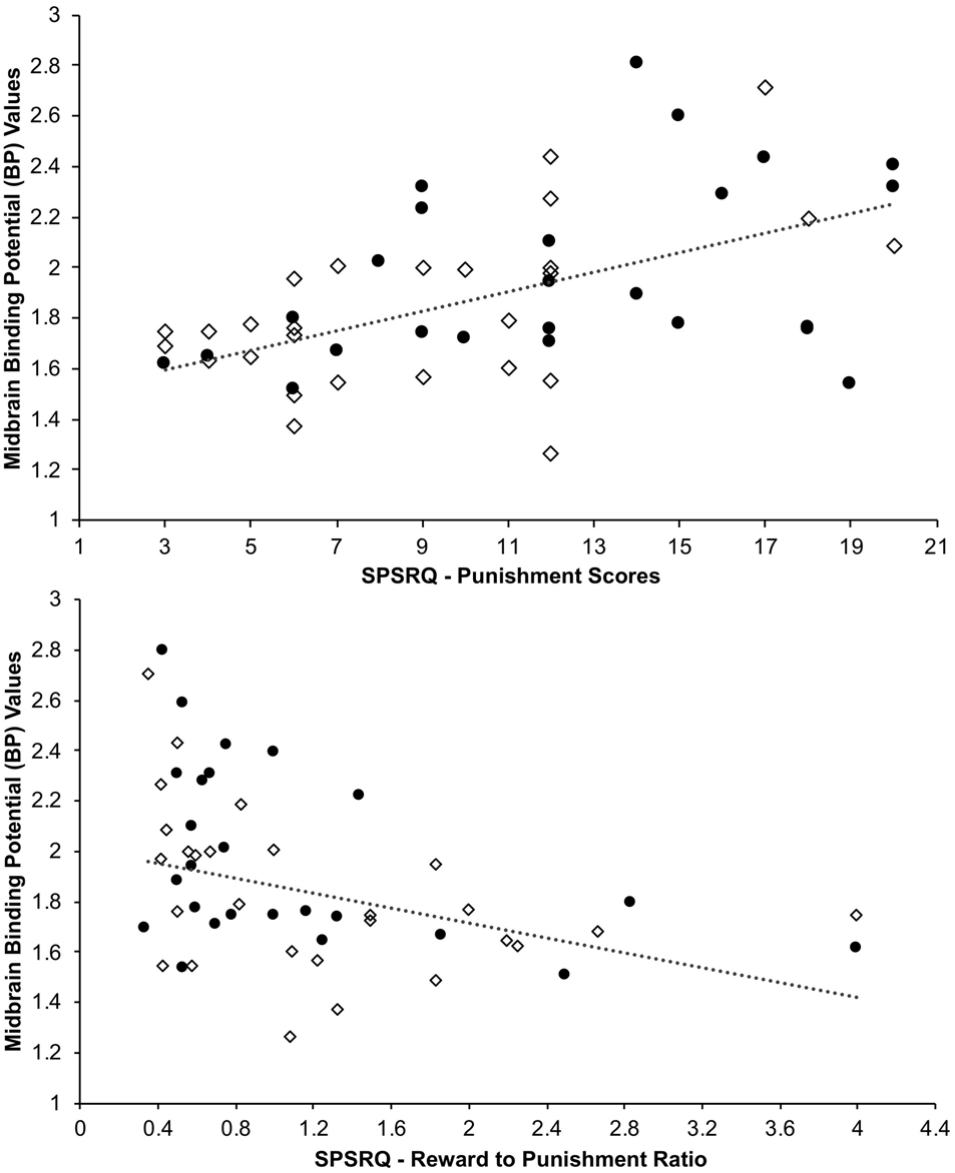
**Upper:** Correlation between midbrain BP_ND_ values and Sensitivity to Punishment scores across the entire cohort (rho = 0.51, *p* = 0.00014, *N* = 52; circles: high EXT; diamonds: low EXT). **Lower:** Correlation between midbrain BP_ND_ values and Sensitivity to Reward:Punishment score ratios across the entire cohort (*rho* = −0.47, *p* = 0.00042, *N* = 52; circles: high EXT; diamonds: low EXT).

Across the whole sample, there was a positive correlation between midbrain BP_ND_ values and SP scores (*rho* = 0.51, *p* = 0.00014, *N* = 52; partial correlation: *r* = 0.51, *p* = 0.0002, *N* = 46; Fig. 3). When the high and low EXT groups were analyzed separately, the correlations did not survive our conservative statistical correction, but the same associations were evident at the trend level (low EXT: *rho* = 0.54, *p* = 0.003; high EXT: *rho* = 0.43, *p* = 0.046). Exploratory analyses yielded a negative correlation between midbrain BP_ND_ values with the SR:SP ratio (*rho* = −0.47, *p* = 0.00042, *N* = 52; partial correlation: *r* = −0.37, *p* = 0.010, *N* = 46; Fig. 3). No other correlations between variables of interest (SURPS, BIS, SPSRQ), and BP_ND_ values were significant at our threshold of *p* < 0.0007.

## Discussion

We measured extra-striatal DA_2/3_R availability in well-characterized youth who had been followed since birth. Participants were tested during their transition years to adulthood, a period of significant fronto-cortical pruning [46] and mesocortical DA pathway expansion [47]. It is also the developmental stage when drug and alcohol experimentation is most likely to escalate to problematic use. As expected, participants at elevated risk for SUDs, based on higher pre-existing EXT traits, had more lifetime occasions of drug use and binge drinking, a denser history of lifetime psychiatric issues, and higher AUDIT, Impulsivity, BIS-11 and SR scores than those with low EXT traits. More novel is the finding that the high-risk participants exhibited elevated [^18^F]fallypride BP_ND_ values throughout the PFC and limbic and paralimbic regions, even after controlling for drug and alcohol use. These widespread increases in terminal region DA_2/3_R availability could reflect low levels of competing extracellular DA or greater extra-striatal DA_2/3_R density. Since reductions in extracellular DA do not yield pronounced effects on extra-striatal [^18^F]fallypride BP_ND_ values [48], the latter interpretation may be more plausible. Group differences were not evident in the midbrain cell body region, but, across the entire sample, midbrain DA_2/3_R availability correlated positively with SP scores.

Individual differences in cortical DA projections might play particularly important roles in the development of SUD susceptibility related phenotypes [47]. Cortical DA transmission sustains neural activity, facilitating the planning of complex behavioral sequences, impulse control, and reward processing [11, 17]. Though most attention has been paid to cortical DA_1_ receptors, accumulating evidence indicates that DA_2/3_Rs also modulate cortical cell excitability, influencing perseverative behaviors and behavioral inhibition, perhaps through actions on subcortical projection sites [11, 49]. The elevated cortical DA_2/3_R availability in our high-risk youth might aggravate susceptibility to these maladaptive behaviors, as greater PFC DA_2/3_R-mediated neurotransmission has been associated with impulsive behaviors in laboratory animals [50].

Group differences were also observed in limbic and paralimbic regions. The elevated amygdalar DA_2/3_R availability in high EXT participants may lead to difficulties in emotion regulation [15] and altered appetitive responses to cues associated with drugs and other rewards [51]. However, the contribution of these DA_2/3_Rs might be complex: in laboratory animals, their stimulation decreases impulsive behaviors and incentive motivational effects [52, 53], while also increasing cocaine-seeking behaviors [54]. The insula is increasingly implicated in diverse addiction-related processes, including emotional aspects of risky decision making [55], and drug craving [56]. Indeed, compared with healthy controls, people with an alcohol use disorder have been reported to exhibit lower baseline insular DA_2/3_R BD_ND_ values [57].

The mechanism by which the high-risk, high EXT participants came to have elevated DA_2/3_R availability is unknown, and cannot be answered from our study alone. However, it is unlikely to reflect diminished competition from extracellular DA since experimentally induced decreases in DA release have minimal effects on [^18^F]fallypride binding [48]. One possibility is that increased extra-striatal DA_2/3_R levels reflect an adaptation to chronically low extracellular DA levels. Alternatively, the greater density of DA_2/3_R in high EXT individuals may be an inherited feature; indeed, the prior evidence of genetic predispositions to low DA_2_ receptor function in addiction susceptible populations has now been shown to reflect biased allele frequencies in healthy control samples [58]. In either scenario, the widespread increases in DA_2/3_Rs could lead to elevated DA_2/3_R-mediated signaling, disrupting better calibrated reward processing [59, 60].

The [^18^F]fallypride BP_ND_ values in our low-risk participants (see unadjusted BP_ND_ values in Supplementary Table 1) were similar to what have been previously found in a large sample of healthy volunteers [61]. However, the BP_ND_ values in our adolescent sample overall were generally higher than what has been observed in middle-aged adults [61], consistent with DA_2_ receptor expression declines with increased age [61, 62]. Of potentially greater importance, the [^18^F]fallypride BP_ND_ values in our high-risk participants and age-matched controls [61] were higher than what we have found in people with a current moderate to severe SUD [17, 51], particularly in limbic and paralimbic regions (Supplementary Table 1). It remains unknown whether a switch from high to low DA_2/3_R availability occurs in high EXT individuals who transition to a SUD, but this is plausible given that a compensatory down-regulation in receptor density has been reported in nonhuman primates following frequent drug-induced surges in extracellular DA [63]. These hypothesized decrements have been proposed to contribute to the diminished incentive value of nondrug rewards and the progressive narrowing of interests [4, 64].

The last major finding in the present study was the relation between sensitivity to punishment and midbrain DA_2/3_R availability, plausibly reflecting the degree of somatodendritic autoreceptor-mediated inhibitory feedback [7, 17, 18]. Of note, lower striatal DA reactivity, which has been linked to increased levels of midbrain D_2/3_ autoreceptors, has been associated with higher trait anxiety [65]. The absence of a group difference in midbrain DA_2/3_R availability highlights how the EXT risk pathway can be combined with varying features, either additional impulsivity-related traits (e.g., low punishment and high reward sensitivity) or anxious, internalizing traits (e.g., high punishment sensitivity). Both might be associated with elevated terminal region DA_2/3_R availability yet high vs. low mesostriatal DA reactivity, respectively.

### Strengths and limitations

The current study has multiple strengths. This includes the use of high-resolution PET imaging in a relatively large sample (for PET studies) of well-characterized participants with a narrow age range prospectively followed from birth. Nevertheless, some limitations exist. First, the imaging data are cross-sectional, and we do not know whether they will identify future substance use problems. Second, our PET scans lasted 90-min, which is suitable for measuring DA_2/3_R availability in the targeted extra-striatal regions but not the striatum, which requires a longer scan [42]. This noted, a large body of work indicates that striatal DA_2/3_R availability is not decreased (and tends to be increased) in people at familial risk for SUDs [66]. Here, we report, to our knowledge, the first evidence of altered extra-striatal DA_2/3_R availability in transitional aged youth at risk for SUDs. Third, some have interpreted elevated DA_2/3_R availability as a protective feature [67, 68]. The elevated DA_2/3_R availability observed in the current study might also reflect this. However, the prospectively documented risk traits and behaviors, including substance use problems already, argues against the protection hypothesis, and more strongly favors the risk hypothesis. Fourth, group differences were seen in terminal region DA_2/3_R availability, but the individual differences in BP_ND_ did not significantly correlate with clinical features or other behavioral traits. This might indicate that complex behavioral effects of extra-striatal terminal region DA transmission emerge from cumulative actions at multiple receptors. In comparison, midbrain DA_2/3_R availability correlated with SP scores, raising the possibility that these putative autoreceptors are more closely related to overall mesostriatal DA function and mesostriatal DA-related behaviors [7, 17, 18]. Fifth, sex was imbalanced in both EXT groups. Exploratory analyses of the BP_ND_ data suggest that the effect of group might be stronger in males than females, but there was neither a main effect of sex nor a sex by risk group interaction (data not shown). Given the relatively small subsamples, it is possible that sex-specific effects exist and should be further tested in future work. Finally, individuals with high EXT traits had a denser history of mental health problems. However, the inclusion of any past or present psychiatric illness (*N* = 14) as an additional covariate did not alter the group differences in BP_ND_ values (data not shown); the same is true for the addition of current SUDs (*N* = 5; mainly cannabis related [*N* = 4]). Indeed, the presence of clinically relevant symptoms prior to the onset of SUDs has been considered an expression of the developing risk pathway [1, 2, 5].

## Funding and disclosure

This work was supported by grants from the Canadian Institutes for Health Research (CIHR): MOP-133537 (ML, JRS, CB, MB), MOP-44072 (JRS), MOP-97910 (JRS, SP); from the Fonds de Recherche du Quebec (FRQ)-Santé: 981055, 991027 (JRS), 35282 (NCR); FRQ-Société et Culture: 2002-RS-79238, 2009-RG-124779 (JRS, MB); from the Social Sciences and Humanities Research Council of Canada (SSHRC): 410–99–1048, 839–2000–1008 (JRS, MB). The QLSCD cohort born in 1997–1998 is led by the Institut de la Statistique du Québec, in collaboration with several departments and agencies of the Government of Quebec and collaborating researchers, including authors of this article. Project completion with QLSCD participants was authorized by the QLSCD Steering Committee. None of the authors have conflicts of interest to disclose.

## Supplementary information

Supplementary Material

Supplementary Figure 1
