# Supplementary material for: Extra-striatal D2/3 receptor availability in youth at risk for addiction
Source: Neuropsychopharmacology. 2020 Apr 7;45(9):1498–505. doi: 10.1038/s41386-020-0662-7 (PMC7360619; doi:10.1038/s41386-020-0662-7)
Supplement: Supplementary file 1 — Supplementary Material [file 41386_2020_662_MOESM1_ESM.docx]

**SUPPLEMENTARY MATERIAL**

**Supplementary Figure 1:** Regions of interest (ROIs) used in the current study.

**Supplementary Table 1:** Raw Fallypride BP_ND_ Values in Regions of Interest in High and Low Externalizing (EXT) Trait Score Youth (means ± standard errors of the mean)

| **Regions of Interest** | **High EXT Group**  **(N=27)** | **Low EXT Group**  **(N=31)** |
| --- | --- | --- |
| Superior frontal gyrus | 0.68±.059 | 0.56±.030 |
| Middle frontal gyrus | 0.83±.063 | 0.70±.024 |
| Medial frontal gyrus | 0.77±.055 | 0.67±.029 |
| Orbitofrontal gyrus | 1.04±.050 | 1.00±.042  (N=30; 1 outlier removed) |
| Hippocampus | 1.48±.058  (N=26; 1 outlier removed) | 1.48±.047  (N=29; 2 outliers removed) |
| Amygdala | 3.22±.12  (N=26; 1 outlier removed) | 2.85±.10  (N=30; 1 outlier removed) |
| Insula | 1.67±.082  (N=26; 1 outlier removed) | 1.55±.066  (N=29; 2 outliers removed) |
| Midbrain | 1.94±.073  (N=26; 1 outlier removed) | 1.85±.064  (N=28; 2 outliers removed/1 file unavailable) |
