# Supplementary figures and images for: Extra-striatal D2/3 receptor availability in youth at risk for addiction
Source: Neuropsychopharmacology. 2020 Apr 7;45(9):1498–505. doi: 10.1038/s41386-020-0662-7 (PMC7360619; doi:10.1038/s41386-020-0662-7)

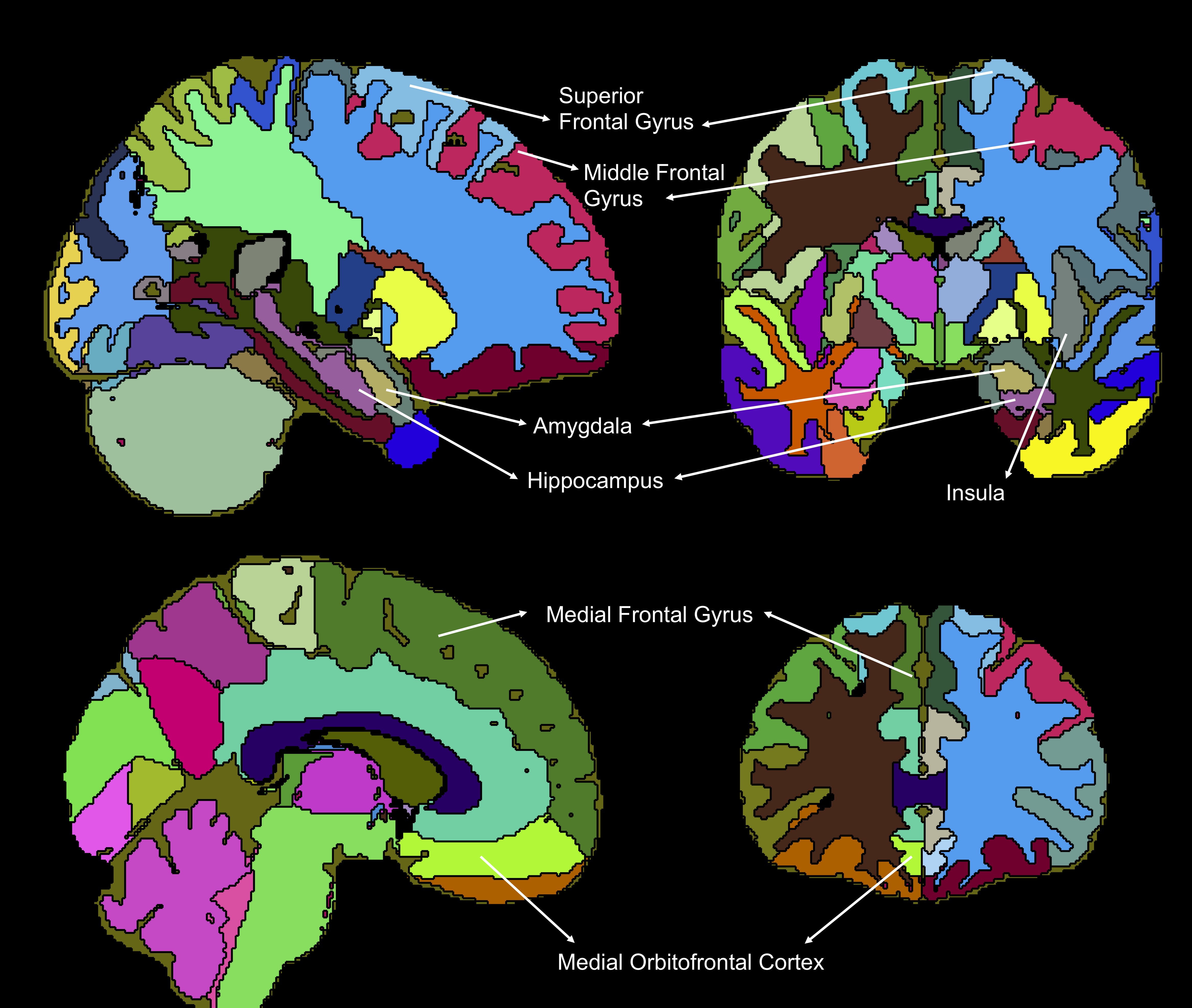

Supplement: Supplementary file 2 — Supplementary Figure 1 [file 41386_2020_662_MOESM2_ESM.jpg]
